# Whole Blood Viscosity Reference Intervals and Its Correlation with Hematology and Serum Chemistry in Cats Using Scanning Capillary Method

**DOI:** 10.3390/ani13233694

**Published:** 2023-11-29

**Authors:** Chae-Yeong Lee, Sung-Lim Lee, Eunju Kim, Jinsu Kang, Sunjun Jung, Namsoo Kim, Jinmu Jung, Dong Hwan Lee, Yoon-Ho Roh, Dongbin Lee

**Affiliations:** 1 Institute of Animal Medicine, College of Veterinary Medicine, Gyeongsang National University, Jinju 52828, Republic of Korea; dlcodud0924@naver.com (C.-Y.L.); sllee@gnu.ac.kr (S.-L.L.); yoonhoroh@gnu.ac.kr (Y.-H.R.); 2 Division of Animal Diseases & Health, National Institute of Animal Science, Rural Development Administration, Wanju 55654, Republic of Korea; keunjunim@korea.kr; 3 Department of Veterinary Medicine and Surgery, College of Veterinary Medicine, University of Missouri, Columbia, MO 65211, USA; jknf5@umsystem.edu; 4 College of Veterinary Medicine, Jeonbuk National University, Iksan 54596, Republic of Korea; vet2010@naver.com (S.J.); namsoo@jbnu.ac.kr (N.K.); 5 Division of Mechanical Design Engineering, College of Engineering, Jeonbuk National University, Jeonju 54896, Republic of Korea; jmjung@jbnu.ac.kr (J.J.); 0311dhlee@jbnu.ac.kr (D.H.L.)

**Keywords:** whole blood viscosity, reference interval, shear rate, scanning capillary tube viscometer, cat

## Abstract

**Simple Summary:**

Blood viscosity, an essential hemorheological parameter, signifies the inherent resistance encountered during blood circulation within blood vessels. Assessing whole blood viscosity stands as an early diagnostic modality for an array of vascular conditions, encompassing cardiovascular, cerebrovascular, and microvascular ailments. Given the intricate challenge of early-stage diagnosis using conventional blood tests, blood viscosity emerges as a tool to gauge disease susceptibility. Recently, scanning capillary viscometers that can obtain blood viscosity values for a wide range of shear rates through a single measurement have been widely used. Using this, this study aimed to establish reference intervals in cats, representative companion animals, for further hemorheological study and the veterinary clinical field. The outcomes of this study yield essential reference intervals for normal whole blood viscosity in healthy feline subjects, encompassing a wide range of shear rates that hitherto lacked comprehensive establishment.

**Abstract:**

Whole blood viscosity, a hemorheological factor, is currently used for diagnosis, as it is correlated with various vascular diseases that are difficult to diagnose early with a general blood test. It was determined that it was necessary to set reference intervals for further studies and utilization of whole blood viscosity in cats, a representative companion animal, and this study was conducted. Fifty healthy cats were recruited for the study, and whole blood viscosity, complete blood count, and serum chemistry tests were performed. The reference intervals of whole blood viscosity were 15.169 to 43.684 cP at a shear rate of 1 s^−1^ reflecting diastole, and 3.524 to 5.544 cP at a shear rate of 300 s^−1^ reflecting systole. Red blood cells, hematocrit, hemoglobin, white blood cells, and neutrophils in the complete blood count, and total protein, albumin, globulin, and cholesterol in the serum chemistry were significantly correlated with whole blood viscosity. The results of this study set the reference intervals of whole blood viscosity for healthy cats in a wide shear rate range that has not yet been fully established, and its correlation with other blood indicators investigated.

## 1. Introduction

Hemorheology is the study of the flow and deformation of blood and its elements in plasma and cells [1]. Whole blood viscosity (WBV), a representative hemorheological factor, is the inherent resistance of blood to flow and represents the thickness and stickiness of blood. The shear rate (SR) is the ratio of fluid velocity to lumen diameter. Whole blood is a non-Newtonian fluid that gradually decreases as the shear rate increases and is a shear-thinning fluid. The major factors that determine WBV are the hematocrit, red blood cell (RBC) aggregation, RBC deformability, and plasma viscosity [2]. The flow rate of blood changes during a cardiac cycle, diastole and systole, and the shear rate continues to change accordingly. The blood becomes stickier when it moves slowly during diastole and thinner when it moves quickly during systole [2]. The WBV continues to change as the shear rate changes; thus, measuring the accurate WBV for the continuously changing shear rate is important [3].

Conventional methods for measuring WBV include a rotating viscometer, scanning capillary tube viscometer (SCTV), falling ball viscometer, and falling needle viscometer [4]. A rotating viscometer, which has been used for a long time worldwide, can only measure viscosity for one shear rate at a time and the measurable shear rate is limited. Consequently, multiple experiments are necessary to evaluate WBV over several shear rate ranges. The U-shaped SCTV in the present study overcomes these disadvantages. Short measurement times and WBV measurements over a wide range of shear rates offer the advantages of efficient and accurate measurement, along with being safe from the risk of infection due to disposable U-tubes [5]. To predict the risk of disease through blood viscosity, it is necessary to accurately measure blood viscosity for continuously changing shear rates. 

An increase in WBV causes disorders in blood flow. WBV has been reported to be independently correlated with well-known cardiovascular risk factors, such as hyperlipidemia, diabetes, hypertension, obesity, cigarette smoking, male sex, and aging in humans [6,7]. Elevated concentrations of gamma globulins, whether monoclonal in malignant diseases or polyclonal, often associated with rheumatic disorders, represent the primary cause of hyperviscosity. Conditions such as polycythemia vera, characterized by an excess of red blood cells, can also contribute to increased blood viscosity. Additionally, hyperviscosity may arise from significant elevations in both mature and immature white blood cell concentrations [8]. Hyperviscosity syndrome is also a typical viscosity disorder that causes vision abnormalities, neurological symptoms, and heart failure due to abnormal hemodynamic properties such as polycythemia, leukemia, and thrombocytosis [8]. WBV is used as an early diagnostic factor for cardiovascular and neurological diseases, such as atherosclerosis and stroke. The risk of such disease increases with elevated WBV, representing a potential application of WBV as a biomarker to predict the risk and severity of ischemic cardiovascular diseases in conjunction with conventional cardiac risk factors [6,9]. It is also used as a factor for evaluating the prognosis of secondary complications caused by hyperviscosity syndrome or microcirculation disorders, such as diabetes and glaucoma. Since early diagnosis of cardiovascular disease and hyperviscosity syndrome is difficult with only general biochemical blood tests, the risk of various cardiovascular diseases can be evaluated using blood viscosity, which is a physical characteristic of blood.

While research on WBV is active in human medicine, research on blood viscosity in veterinary medicine is insufficient, despite frequent cardiovascular and cerebrovascular diseases in animals. Hemodynamic approaches are also necessary for diseases such as cardiovascular aortic thrombo-embolism and laminitis. Previous studies have been conducted on the measurement of total blood viscosity, plasma viscosity, and RBC aggregation in nine mammals [10] and on normal reference values in dogs [11]; however, studies on these reference intervals (RIs) are insufficient in cats.

Therefore, this study attempted to establish RIs for WBV, and investigate the correlation between WBV and blood tests using a U-shaped SCTV, which can obtain results from a wide range of shear rates (1–1000 s^−1^).

## 2. Materials and Methods

### 2.1. Animal Preparation

Fifty healthy client-owned cats were recruited for the study. General physical examination, hematology, and complete biochemistry were performed to ensure that the patients did not have any underlying diseases. Exclusion criteria were a history of current disease, abnormalities detected on physical examination, or significant abnormalities in the recent complete blood count (CBC) or serum biochemistry examination including albumin (ALB), alkaline phosphatase (ALKP), alanine transaminase (ALT), amylase (AMYL), blood urea nitrogen (BUN), creatinine (CRE), gamma-glutamyl transferase (GGT), glucose (GLU), and total protein (TP). All cats in the study had no history of surgery other than neutering or spaying, and were not being administered any medications at the time of participation. Food, but not water, was withheld for a minimum of 6 h before the start of the blood test. 

### 2.2. Blood Sample Collection

Blood samples were collected (5 mL) from the jugular vein using disposable syringes and 23-gauge needles. Each blood sample was divided into three standard tubes, which were EDTA-coated tubes for CBC and WBV and a heparin tube for chemistry. Blood tests were performed immediately after collection. Heparinized plasma, separated from the collected blood in heparin tubes, was centrifuged at 5500 rpm for 5 min.

### 2.3. Whole Blood Viscosity, Hematological, and Serum Chemical Analysis

WBV was measured using the Casson model U-shaped SCTV (Rheovis-01; Biorheologics Co., Ltd., Jeonju, Republic of Korea). The blood flows into a U-shaped capillary tube, filling one vertical column higher than the other, followed by equilibration through gravity forces. Initially rapid, the flow gradually slows until the blood levels in the two connected capillary tubes are nearly equal. In other words, the shear rate automatically changes from high to very low, and the shear rate is automatically measured at every moment, resulting in the ability to measure viscosity across all shear rate ranges. While the blood level equilibrates, the system records each column’s height over a defined 3 min period. The velocity of blood flow, dictated by the rate of height change in the blood columns in the tube, is directly associated with the pressure drop across the capillary tube. This relationship facilitates the automatic calculation of shear rate, shear stress, and WBV for the blood sample. Regarding the calculation of the viscosity (μ), the viscosity is mathematically calculated as the ratio of wall shear stress (τ_w_) to wall shear rate (γ_w_), as described by the following equation: μ = τ_w_/γ_w_. The U-shaped SCTV in this study calculated WBV values at shear rates ranging from 1 s^−1^ to 1000 s^−1^, and within that range of shear rates, it provided representative WBV values according to shear rates of 1, 5, 10, 50, 100, 150, 300, and 1000 s^−1^.

CBC was measured using an automatic blood cell counter (IDEXX ProCyte Dx^®^ Hematology Analyzer; IDEXX Laboratories, Inc., Westbrook, ME, USA). The red blood cell count (RBC, 1012/L), hemoglobin concentration (HGB, g/dL), hematocrit (HCT, %), white blood cell count (WBC, 109/L), platelet count (PLT, 109/L), mean corpuscular volume (MCV, fL), mean corpuscular hemoglobin (MCH, pg), mean cell hemoglobin concentration (MCHC, g/dL), red cell distribution (RDW, %), and mean platelet volume (MPV, fL) were measured.

Serum chemistry was measured using an automatic chemistry analyzer (Catalyst One^®^ Chemistry Analyzer, IDEXX Laboratories, Inc., Westbrook, ME, USA). The concentrations of ALB (g/dL), ALKP (U/L), ALT (U/L), AMYL (U/L), BUN (mg/dL), CRE (mg/dL), GGT (U/L), GLU (mg/dL), TP (g/dL), calcium (Ca, mg/dL), cholesterol (CHOL, mg/dL), globulin (GLOB, g/dL), lipase(LIPA, U/L), phosphorus (PHOS, mg/dL), and total bilirubin (TBIL, mg/dL) were measured.

### 2.4. Statistical Analysis

The RIs of WBV in cats were established using the “Reference Value Advisor”, Microsoft Excel-based freeware, following the American Society for Veterinary Clinical Pathology (ASVCP) guidelines [12]. Since the number of cats was 50 and the Shapiro–Wilk test satisfied normality, the reference limit was set to 90% confidence interval, and the data were presented as the mean and standard deviation (SD). To evaluate the differences between sexes and breed of WBV in cats, the differences were compared using a one-way analysis of variance (ANOVA). Pearson’s correlation analysis was used to determine the correlation between WBV and conventional blood tests, including hematology and serum chemistry. SPSS 27.0.0 (PASW Statistics; IBM Co., Armonk, NY, USA) and GraphPad Prism 8.0.2 (GraphPad Software, Inc., San Diego, CA, USA) were used for statistical analysis. *p*-values were considered statistically significant at *p* < 0.05.

## 3. Results

The reference population consisted of 50 healthy cats. Domestic Korean Short hair (*n* = 30), American Short hair (*n* = 3), Turkish Angora (*n* = 4), Siamese (*n* = 3), mixed cats (*n* = 2), Persian (*n* = 2), Russian blue (*n* = 1), Scottish fold (*n* = 2), Bengal (*n* = 1), British long hair (*n* = 1), and Ragdoll (*n* = 1) were included. There were 29 male (25 castrated) and 21 female (18 spayed) cats in this study. Cats, 0.6–10 years (median 4 years) and weighing 3–8 kg (median 4.75 kg), were included in this study. Statistically, differences between WBV and sex or breed were not observed in the results.

### 3.1. Reference Intervals of WBV

**Figure 1:**
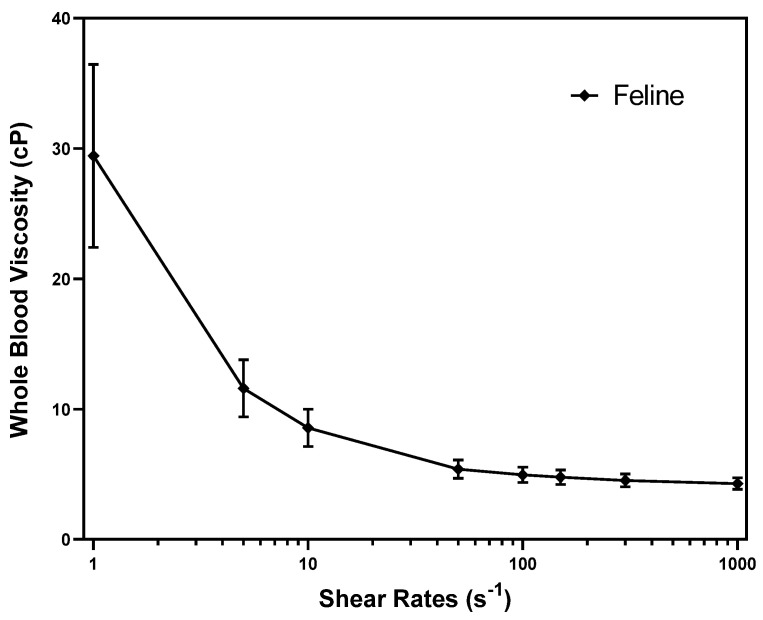
Mean whole blood viscosity (cP) at the shear rates from 1 to 1000 s^−1^ in cats. The graph shows the mean value with the standard deviation (error bars).

The RIs of WBV in cats are presented in Table 1 and Figure 1. The representative RIs of WBV based on the shear rates of 1, 5, 10, 50, 100, 150, 300, and 1000 s^−1^ in cats are presented (Table 1 and Figure 1). WBV at a low shear rate (1 s^−1^) is defined as diastolic WBV, and at a high shear rate (300 s^−1^) as systolic WBV [5]. The RIs of diastolic WBV were 15.169–43.684 (cP) and the systolic WBV was 3.524–5.54 (cP). The graph of the mean value with the standard deviation shows that the WBV decreased as the shear rate increased (Figure 1).

### 3.2. Correlation between WBV and Hematology

**Figure 2:**
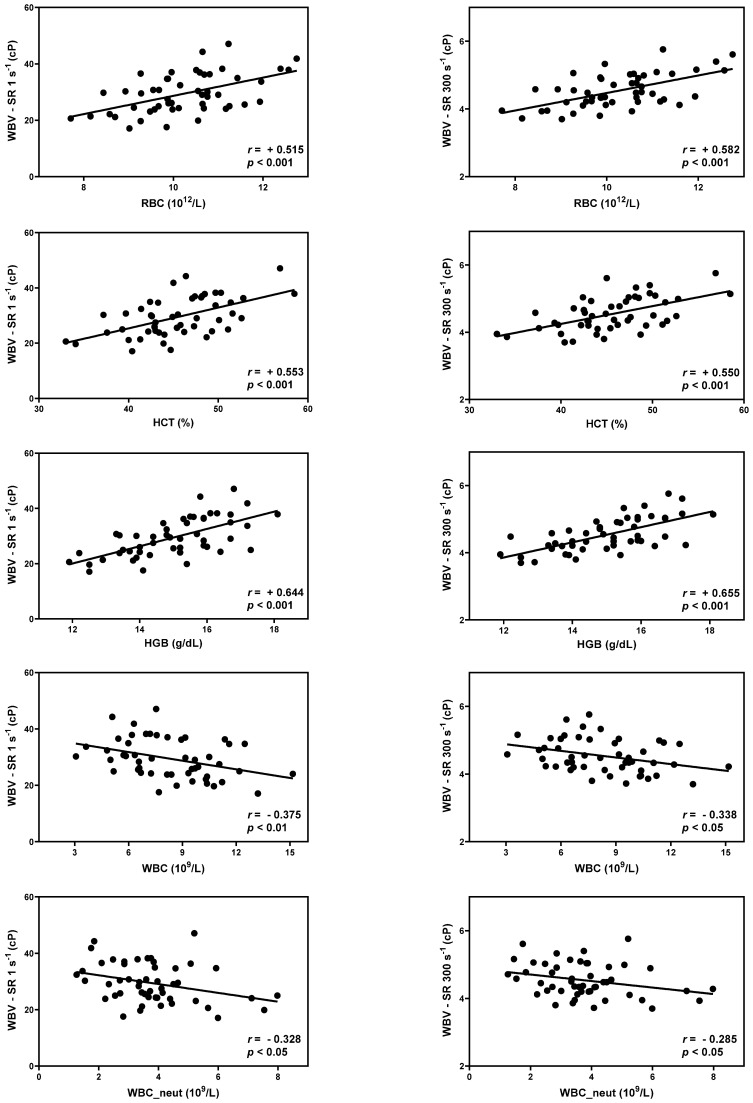
Scatter diagram with r-values between whole blood viscosity and hematology at the shear rate 1 s^−1^ (diastole) and 300 s^−1^ (systole).

**Figure 3:**
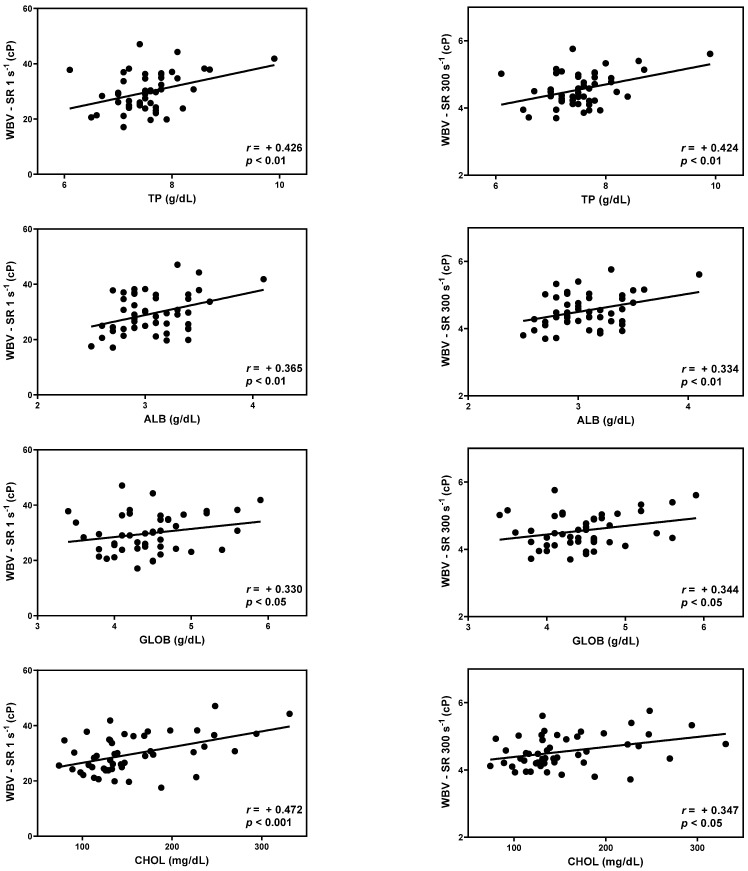
Scatter diagram with r-values between whole blood viscosity and total protein, albumin, globulin, and cholesterol at the shear rate 1 s^−1^ (diastole) and 300 s^−1^ (systole).

Correlation analysis was performed using Pearson’s correlation coefficient. The r-values are described in Table 2 and Table 3, and scatter diagrams with the r-values of the correlation values are presented (Figure 2 and Figure 3). The r-values were evaluated by dividing it into five sections: poor (0 < r < ±0.3), fair (±0.3 ≤ r < ±0.6), moderate (±0.6 ≤ r < ±0.8), very strong (±0.8 ≤ r < ±1), and perfect (±1), following the reference guidelines [13,14].

RBC, HCT, HGB, and WBC counts were statistically correlated with WBV over the entire range of shear rates. Neutrophils were statistically correlated with WBV at shear rates ranging from 1 to 300 s^−1^ (Table 2). The correlation coefficient values (r) of RBC and HCT showed a positive and fair correlation, while HGB showed a positive and moderate correlation. (*p* < 0.001) The correlation coefficient values (r) of WBC show negative and fair correlation, and neutrophils show negative and poor-to-fair correlation (*p* < 0.05). Scatter diagrams with r-values between WBV at a shear rate of 1 s^−1^ (diastole) and 300 s^−1^ (systole), and RBC, HCT, HGB, WBC, and neutrophil counts are presented (Figure 2).

### 3.3. Correlation between WBV and Serum Chemistry

TP, ALB, GLOB, and CHOL were significantly correlated with WBV over the entire range of shear rates (Table 3). The correlation coefficient values (r) for TP, ALB, GLOB, and CHOL showed positive and fair correlations. (*p* < 0.05) Scatter diagram with r-values between WBV at shear rate of 1 s^−1^ (diastole) and 300 s^−1^ (systole), and TP, ALB, GLOB, and CHOL are presented (Figure 3).

## 4. Discussion

The present study is the first report of the RIs of WBV in healthy cats using a U-shaped SCTV over a wide range of shear rates from 1 to 1000 s^−1^. As a result, a continuous change in WBV and the characteristics of the shear-thinning fluid are indicated. The mean WBV of the present study in cats reflects the characteristics of shear-thinning fluid.

In this study, none of the sexes and breeds were statistically different in WBV. In human medicine, males have higher WBV, HCT, fibrinogen concentration, and RBC aggregation, and lower RBC deformability than females. Physiological changes, such as the menstrual cycle, pregnancy, or the production of sex hormones, can affect these hematological parameters in humans [15,16,17,18,19]. HCT, one of the major determinants of WBV, is known to be lower in female dogs and cats than in males [20,21]. However, in our study, castrated males were 25/29 and spayed females were 18/21. Therefore, it is considered to minimize sex-related effects. In addition, since the statistical significance of breed did not have differences in WBV, it is important to set the RIs through results from the 50 cats used in this study, regardless of the sex or breed.

The RIs of WBV in cats were previously measured at three different shear rates (0.7, 2.4, and 94 s^−1^) using a rotational viscometer. All data in this previous study are presented as the medians with the 25th and 75th percentiles. From the study, each WBV at 0.7 s^−1^, 2.4 s^−1^, and 94 s^−1^, the medians (25th/75th percentiles) were 30.194 (26.899/33.971), 15.349 (13.871/17.251), and 4.442 (4.183/4.671) cP with 40% HCT in variable breeds of cats [10]. In our results, the calculated median (25th/75th percentiles) WBVs at 0.7 s^−1^, 2.4 s^−1^, and 94 s^−1^ were 37.141 (30.313/45.72 4), 16.732 (14.277/20.134), and 4.855 (4.520/5.502) cP. As mentioned above, the low shear rate reflects the blood flow of diastole, and the high shear rate reflects the systole. For a clinical comparison, a low shear rate is used as 5 s^−1^ or less, and a high shear rate is used as 300 s^−1^ [2,15]. Therefore, despite similar results in both studies, the RIs of the previous study were limited in that the WBV at the systole was not measured, while our results established the RIs of WBV in a wide range of shear rates. Examining WBV across a broad spectrum of shear rates can yield a precise comprehension of the variations in WBV corresponding to different shear rates.

The correlation between WBV and hematology was similar to that reported in many previous studies [2,10]. Increased HCT disrupts blood flow, increases WBV, and in addition to the concentration of RBC, the rheological properties of cells, such as aggregation and deformation, have an important influence on blood flow. It is generally accepted that RBC aggregation is a major factor contributing to the increase in WBV at low shear rates. If blood flows very slowly at a low shear rate, rouleaux formation occurs in the RBC and the flow resistance force increases WBV. At a high shear rate, the aggregated RBCs are scattered individually and distributed evenly in the blood, lowering the flow resistance, which significantly reduces WBV. Increased shear rates interfered with aggregation, whereas reduced shear rates tended to cause aggregate of RBCs [22]. One of the characteristics of RBC is their high deformability. Deformability describes the ability of an RBC to change its shape, involving cell curvature, uniaxial deformation, or area expansion, in response to a deforming force [23]. If the deformability is impaired, it does not aggregate well even at a low shear rate, which does not significantly affect WBV. Conversely, at a high shear rate, it limits the cell orientation in the flow and increases WBV. Thus, the RBC has a significant effect on WBV. In our study, the RBC, HCT, and HGB were statistically correlated with WBV over the entire range of shear rates.

However, the relationship between WBV and WBC has not been fully studied. WBCs have a negligible effect on WBV than RBC, however, especially in microcirculation, where blood vessel sizes are even smaller than the size of blood cells, they may have the potential to influence flow [24]. The relationships between WBC count and WBV has been analyzed in human medicine. They proved that both RBC and WBC can affect the WBV [25]. WBC was statistically correlated with WBV over the entire range of shear rates in our study.

In the present study, the main correlated factors were plasma proteins. Plasma proteins are composed of albumin, globulin, and fibrinogen, and WBV is directly or indirectly affected by plasma proteins through their molecular weight and influence on RBC aggregation. Hyperviscosity syndrome can also be caused by abnormalities in plasma proteins [8,26]. There is evidence of a correlation between TP and WBV in cats [10]. Moreover, correlations among TP, GLOB, CHOL, and WBV were found in dogs [11]. TP was statistically correlated with WBV over the entire range of shear rates in the cats in this study. ALB is the most abundant protein in the blood plasma. There was no correlation between ALB and WBV in dogs [11], but it was statistically correlated over the entire range of shear rates in this study. The main components of the globulin fraction are immunoglobulins, which are produced by lymphoid tissues in response to antigenic stimulation. Several rheological disorders are associated with abnormalities in plasma immunoglobulins. Hyperviscosity syndrome is often present and is caused by immunoglobulins depending on their concentration, molecular weight, and molecular size [8]. Multiple myeloma, a multifocal plasma cell neoplasm, is rare in cats, with an estimated incidence of <1% of all feline hematopoietic neoplasms. However, hyperviscosity syndrome has been previously reported in cats in which retinal hemorrhages, neurologic signs, or both were noted [27]. Moreover, lymphoma, feline immunodeficiency virus, and feline leukemia virus infection leading to subsequent hypergammaglobulinemia, which is the main cause of hyperviscosity syndrome, have been reported [28,29]. Globulin was statistically correlated with WBV over the entire range of shear rates in our study. 

In human medicine, cholesterol also influences the WBV by affecting RBC aggregation and deformability. The RBC surface has approximately 200 binding sites for LDL or HDL, and there is a distance of 25 nm between the RBCs due to electrostatic repulsions. The diameter of LDL is 18–30 nm, which causes aggregation and rouleaux formation, resulting in an increased WBV. HDL, on the other hand, is not large enough to span between RBCs at 5–12 nm, so it does not cause aggregation, but rather, the binding site acts competitively on LDL to prevent LDL adhesion and WBV from increasing. LDL increases the cholesterol-to-phospholipid ratio of the RBC membrane and reduces its deformability of the RBC membrane [2,30]. In dogs and cats, the size of LDL is reported to be 16–25 nm, and the sizes of the three subtypes are 10–35 nm, 9–12 nm, and 5–9 nm, respectively, with LDL being relatively larger than HDL [31]. Cholesterol levels were statistically correlated with WBV over the whole range of shear rates. 

Psychological stress causes an increase in WBV and hemoconcentration in humans [32]. In cats, glucose and lactate levels rise due to sudden stress [33]. The increase in glucose and lactate levels are known to increase WBV and induce microcirculation. The elevated glucose level increased aggregation and reduced deformability by stiffening the RBC membrane. Furthermore, HCT is elevated in diabetes due to the increased permeability of the capillary vessel wall [34]. Acidosis induced by lactate, pyruvate, or HCl produces RBC swelling, increases the HCT level, and increases WBV [3]. It is thought that this reversible change may contribute to the disorders of microcirculation. Further studies can be conducted to determine whether a temporary rise in these factors in stressful situations causes an increase in WBV and how it affects patients with risk factors of hyperviscosity in cats. In addition, hyperviscosity in humans has been reported to cause cardiovascular disease, microcirculation disorders, and hyperviscosity syndrome. In veterinary medicine, it can also be considered a hematological approach to diseases such as laminitis or aortic thromboembolism, which can cause viscosity abnormalities due to impaired blood flow.

A limitation of this study is that environmental control was not possible because it was not an experiment involving a controlled experimental group of a single breed. In prior studies of human medicine, it was reported that sex differences exist in WBV; thus, sex-specific differences may appear, but there was a lack of a sufficient population in this study. However, further studies are required for each breed. As the ASVCP guidelines specify that the minimum number of populations that do not require a normality test is more than 120, a study in a large controlled experimental group with more than 120 controls for each sex and breed is considered to have clinical significance.

## 5. Conclusions

Our study was conducted to set WBV RIs in 50 healthy cats and suggest correlations between WBV and blood test results in cats. The RIs were 15.169–43.684 (cP) at a low shear rate (1 s^−1^) and 3.524–5.54 (cP) at a high shear rate (300 s^−1^). The results showed a correlation between WBV and RBC, HCT, HGB, and WBC in CBC; and TP, ALB, GLOB, and CHOL in serum chemistry. Since this is the first report of WBV RIs in cats, these reference data would be useful for further veterinary and hemorheological studies.

## Figures and Tables

**Table 1:** Reference intervals of whole blood viscosity in healthy cats (*n* = 50).

	Shear Rate	Descriptive Statistics		RI within 90% CI		
		Mean	SD	RI	Lower Limit	Upper Limit
**Whole Blood Viscosity (cP)**	**SR 1 s^−1^**	29.427	7.025	15.169–43.684	12.574–17.883	40.826–46.423
	**SR 5 s^−1^**	11.591	2.196	7.133–16.048	6.223–8.129	15.092–17.061
	**SR 10 s^−1^**	8.565	1.446	5.630–11.501	5.004–6.258	10.910–12.117
	**SR 50 s^−1^**	5.393	0.707	3.957–6.829	3.675–4.264	6.541–7.106
	**SR 100 s^−1^**	4.955	0.595	3.747–6.163	3.499–4.003	5.901–6.406
	**SR 150 s^−1^**	4.770	0.551	3.653–5.888	3.434–3.877	5.658–6.108
	**SR 300 s^−1^**	4.534	0.498	3.524–5.544	3.302–3.725	5.328–5.729
	**SR 1000 s^−1^**	4.285	0.448	3.375–5.195	3.198–3.574	5.013–5.380

RI, reference interval; CI, confidence interval; cP, centipoise; SR, shear rate; SD, standard deviation.

**Table 2:** Correlation (r-values) between whole blood viscosity and hematology.

	Shear Rates	RBC	HCT	HGB	WBC	WBC_Neut
**Whole Blood Viscosity (cP)**	**SR 1 s^−1^**	0.515 ***	0.553 ***	0.644 ***	−0.375 **	−0.328 *
	**SR 5 s^−1^**	0.521 ***	0.554 ***	0.640 ***	−0.353 *	−0.316 *
	**SR 10 s^−1^**	0.520 ***	0.549 ***	0.632 ***	−0.337 *	−0.306 *
	**SR 50 s^−1^**	0.543 ***	0.538 ***	0.621 ***	−0.329 *	−0.292 *
	**SR 100 s^−1^**	0.565 ***	0.547 ***	0.640 ***	−0.337 *	−0.296 *
	**SR 150 s^−1^**	0.575 ***	0.550 ***	0.649 ***	−0.337 *	−0.292 *
	**SR 300 s^−1^**	0.582 ***	0.550 ***	0.655 ***	−0.338 *	−0.285 *
	**SR 1000 s^−1^**	0.587 ***	0.543 ***	0.658 ***	−0.334 *	−0.275

cP, centipoise; SR, shear rate; RBC, red blood cell; HCT, hematocrit; HGB, hemoglobin; WBC, white blood cell; WBC_neut, neutrophil count. * *p* < 0.05, ** *p* < 0.01, *** *p* < 0.001.

**Table 3:** Correlation (r values) between whole blood viscosity and serum chemistry.

	Shear Rates	TP	ALB	GLOB	CHOL
**Whole Blood Viscosity (cP)**	**SR 1 s^−1^**	0.426 **	0.365 **	0.330 *	0.472 ***
	**SR 5 s^−1^**	0.439 **	0.358 *	0.350 *	0.461 ***
	**SR 10 s^−1^**	0.444 **	0.352 *	0.359 *	0.453 **
	**SR 50 s^−1^**	0.453 **	0.349 *	0.371 **	0.417 **
	**SR 100 s^−1^**	0.442 **	0.344 *	0.360 *	0.390 **
	**SR 150 s^−1^**	0.437 **	0.342 *	0.356 *	0.374 **
	**SR 300 s^−1^**	0.424 **	0.334 *	0.344 *	0.347 *
	**SR 1000 s^−1^**	0.403 **	0.319 *	0.326 *	0.311 *

cP, centipoise; SR, shear rate; TP, total protein; ALB, albumin; GLOB, globulin; CHOL, cholesterol. * *p* < 0.05, ** *p* < 0.01, *** *p* < 0.001

## Data Availability

Data are contained within the article.
